# Efficacy of a Simple Formulation Composed of Nematode-Trapping Fungi and *Bidens pilosa* var. *radiata* Scherff Aqueous Extracts (BPE) for Controlling the Southern Root-Knot Nematode

**DOI:** 10.1264/jsme2.ME17110

**Published:** 2018-03-29

**Authors:** Atsushi Ajitomi, Satoshi Taba, Yoshino Ajitomi, Misa Kinjo, Ken-taro Sekine

**Affiliations:** 1 Okinawa Prefectural Agricultural Research Center 820 Makabe, Itoman city, Okinawa, 901–0336 Japan; 2 Faculty of Agriculture, University of the Ryukyus 1 Senbaru, Nishihara-cho, Nakagami-gun, Okinawa 903–0213 Japan; 3 Okinawa Prefectural, Nanbu Agricultural Development Center 517 Yamakawa, Haebaru town, Shimajiti-gun, Okinawa 901–1115 Japan; 4 Okinawa Prefectural Plant Protection Center 123 Maji, Naha city, Okinawa, 902–0072 Japan

**Keywords:** *Arthrobotrys dactyloides*, *Bidens pilosa* var. *radiata*, mixture formulation, *Meloidogyne incognita*, nematode-trapping fungi

## Abstract

We tested a formulation composed of a mixture of *Bidens pilosa* var. *radiata* extract (BPE) and nematode-trapping fungi for its effects on *Meloidogyne incognita*. In earlier evaluations of the effects of plant extracts on the hyphal growth of 5 species of nematode-trapping fungi with different capture organs (traps), the growth of all species was slightly inhibited. However, an investigation on the number of capture organs and nematode-trapping rates revealed that *Arthrobotrys dactyloides* formed significantly more rings and nematode traps than those of the control. An evaluation of simple mixed formulations prepared using sodium alginate showed that nematodes were captured with all formulations tested. The simple mixed formulation showed a particularly high capture rate. Furthermore, in a pot test, although the effects of a single formulation made from the fungus or plant extract were acceptable, the efficacy of the simple mixed formulation against *M. incognita* root-knot formation was particularly high.

Various species of plant-parasitic nematodes occur worldwide. Root-knot, cyst, and root-lesion nematodes are known to cause serious damage to cultivated crops. Global economic losses due to these nematodes are estimated to be 800 million yen, and damage caused by root-knot nematodes, such as *Meloidogyne incognita*, is particularly significant (27). In addition, nematodes damage the roots of plants, thereby providing an entry point for soil-borne diseases. Chemical control by soil disinfection is used as a control measure; however, this results in the disappearance of useful microorganisms, and causes the disturbance of soil ecosystems. Therefore, implementing measures for nematode control that do not affect the soil environment will result in the suppression of soil-borne diseases.

Integrated pest management (25) and environmentally-based management (18) approaches are gaining acceptance, with the control of agricultural pests without the sustained use of pesticides now being common. Environmentally friendly control approaches include physical, agricultural, and biological methods, which have been employed in studies evaluating solarization (33), reductive disinfection (14, 20), the use of natural enemies, and the utilization of fumigation plants and plant extracts. A number of biological methods to control plant-parasitic nematodes have been evaluated, including *Pasteuria penetrans* (41) and *Streptomyces* sp. (6), nematode-trapping fungi (34, 35), and various plant extracts (5). Taba *et al.* (32) isolated 18 species of nematode-trapping fungi with several capture organs on Okinawa Island and showed that their distribution varied according to vegetation and soil types. They later evaluated the ability to trap the second-stage juveniles (J2s) of *M. incognita* using isolated fungi and screened a fungus exhibiting the highest capture ability (34). These studies (35, 37) also described a combined biological and chemical approach for controlling *M. incognita* using a simultaneous treatment with *Monacrosporium ellipsosporum* and a nematicide. In addition, Taba *et al.* (36) investigated the nematicidal activity of extracts of a weed found primarily on Okinawa Island (*Bidens pilosa* var. *radiata*) on the J2s of *M. incognita* and found that it exhibited potent activity against *M. incognita* and other important nematodes (38). More recently, these authors (39) proposed various methods for the agricultural control of nematodes using plant extracts. In the present study, we examined the effectiveness of combining nematode-trapping fungi and *B. pilosa* extracts (BPE) for controlling nematodes.

## Materials and Methods

### Effects of BPE on the hyphal growth of nematode-trapping fungi

#### Test fungi

Five species of nematode-trapping fungi stored in our laboratory were tested: *Arthrobotrys dactyloides* Drechsler, *A. oligospora* Fresenius, *M. ellipsosporum* (Preuss) R. C. Cooke and Dickinson, *M. eudermatum* (Drechsler) Subramanian, and *M. phymatopagum* (Drechsler) Subramanian.

#### Test plants and aqueous extract

Above-ground tissues from *B. pilosa* var. *radiata* Scherff collected from a subtropical field science center of the University of the Ryukyus were dried using a dry-heat sterilizer (10-630CS; Ikemoto Scientific Technology, Tokyo, Japan) at 100°C for 24 h and then finely chopped. Chopped dried plant tissue (10 g) was extracted with 50 mL of boiling sterile distilled water for 30 min. The resulting extract was filtered through filter paper (no. 2; Advantec, Tokyo, Japan) and used as a stock solution, which was then diluted 10-, 20-, 50-, and 100-fold with sterile distilled water to prepare working solutions.

#### Test medium

Corn meal agar (CMA)-a medium consisted of the following: CMA, 17 g; glucose, 2 g; yeast extract, 1 g; agar, 2 g; distilled water, 1,000 mL. Stock solution plates were prepared by adding 0.1 mL of the stock solution onto each CMA-a plate (diameter of 90 mm) and then spreading it across the surface using a bacterial spreader. On the other hand, CMA-a plates containing *B. pilosa* var. *radiata* extract (BPE) (diluted 10- to 100-fold) plates were prepared by adding the plant extract to an appropriate concentration (1 mL) before the medium solidified. CMA-a, to which 1 mL of sterile distilled water was added, was also used as a control.

### Effects of the plant extract on the hyphal growth of fungi

A colony disk (5 mm) was taken from 2- to 3-week-old colonies of several nematode-trapping fungi pre-cultured on CMA-a using a cork borer and inoculated onto another CMA-a plate containing BPE, which was then cultured at 25°C in an MIR-153 incubator (Sanyo Electric, Osaka, Japan). Since the growth rate differed for each species of fungus examined, the colony diameter (mm) was measured 5–21 d after the inoculation. The experiment was replicated 6 times.

### Effects of BPE on the formation of capture organs and the nematode-trapping rate

#### Test nematode

Single *M. incognita* egg sacs were isolated from eggplant plants (*Solanum melongena* L., cv. Chojya) grown in Nishihara, Uruma, Okinawa, Japan, and cultured on tomato plants (*Lycopersicon esculentum* L., cv. Chibikko) (Marutane, Kyoto, Japan) in a greenhouse without temperature control. Second-stage juveniles (J2s) that hatched from the egg sacs in a Petri dish were used in experiments.

#### Formation of capture organs and the nematode-trapping rate

A circle of 4 cm in diameter was drawn on the back side, central part of a pre-sterilized Petri dish, and 10 mL of CMA-b medium (CMA, 2 g; agar, 16 g; distilled water, 1,000 mL) was added to the dish. After the agar medium had solidified, 0.1 mL of BPE (stock solution and 10-fold dilution) was placed on the medium surface and spread using a bacteria spreader. A cork borer was used to punch out 5-mm disks of the colonies of several nematode-trapping fungi that had been pre-cultured on CMA-b, and the disks were inoculated onto CMA-b plates containing several concentrations of the plant extract. A nematode suspension (200 nematodes 5 mL^−1^) was added to the plate within the area of the circle drawn on the back of the plate, and the plate was cultured at 25°C. The nematode-trapping rate and number of capture organs that formed within the circle were assessed microscopically (Eclipse E200; Nikon, Tokyo) 1, 4, and 7 d after the inoculation. The experiment was replicated 6 times.

### Effects of the formulation composed of BPE and *A. dactyloides* on the formation of capture organs and the nematode-trapping rate

#### Production of the simple mixed formulation

A colony disk was taken from *A. dactyloides* pre-cultured on CMA-b using a cork borer and transferred to an Erlenmeyer flask containing 250 mL of GYM liquid medium (glucose, yeast extract, and malt extract, 1%, respectively). The flask was incubated with shaking (150 rpm) at 25°C for 14 d. After cultivation, the number of fungi was assessed using a hemocytometer (Sunlead Glass, Saitama, Japan); the number of separated partial hyphae by centrifugation in the liquid medium was 2.4×10^5^ mL^−1^. Next, 4% sodium alginate solution was prepared using BPE, 250 mL of this solution was added to the Erlenmeyer flask (500 mL), and an equivalent amount of GYM liquid medium containing partial hyphae was then added, such that the total volume was adjusted to 500 mL. In accordance with the method of Taba *et al.* (35), the simple mixed granular formulation (approximately 2 mm in diameter) was produced using this mixed solution. Four different formulations were produced using this method: liquid medium (GYM), BPE (stock solution, 10-fold dilution), hyphal bodies, and a mixture of BPE (stock solution, 10-fold dilution) and partial hyphae.

#### Formation of capture organs and the nematode-trapping rate

In order to investigate the effects of BPE, 5-granule aliquots of two mixture formulations (stock solution [10-fold dilution] and fungus) were placed at the center on a CMA-b plate. Survival was judged based on hyphal growth evaluated after a 7-d cultivation at 25°C. After 20 d, a suspension of *M. incognita* J2s (200 nematodes 0.5 mL^−1^) was added to the formulations, and the number of nematodes captured was then assessed. Captured nematodes were counted within the 4-cm diameter circle, in which the formulation was placed at the center.

### Efficacy of the simple mixed formulation for controlling *M. incognita*

#### Preparation of test soil

A total of 60 g (equivalent 3.0 t 10 a^−1^) of manure (‘Minori’, Kitanaka yuki SA, containing cattle dung as the main ingredient) as a base fertilizer was mixed with sterile Ryukyu limestone soil ‘Shimajiri mahji’ mixed initially with vermiculite at a ratio of 4:1 (v/v), and approximately 2.5 kg of this prepared soil was placed into 1/5,000 Wagner pots (190 mm depth×159 mm diameter). A 10-mL suspension including approximately 1,000 *M. incognita* J2s was added to each pot and mixed well.

#### Pot test

After the pots were filled with soil, they were fully watered. One of the following formulations (10 g) prepared as described above for Experiment 3 was then added to a hole (*ca.* 5 cm in diameter×5 cm in depth) made in soil:BPE (stock solution) only, fungus only, and the simple mixed formulations (fungus and stock solution of BPE). A 4-week-old ‘Chibikko’ tomato seedling was planted in the hole with one of the formulations. There were five replicates per treatment, and plants were cultivated in a greenhouse without temperature control in the range of approximately 12 to 32°C. Plant height and weight, root weight, root-knot formation, and *M. incognita* population density were measured at the end of the cultivation period after 45 d. The formation of root knots in tomato plants was rated according to the following scale: 0, no root knots; 1, a few (1–2) root knots; 2, a moderate number (3–10) of separated root knots; 3, 11–30 root knots, many of them continuous; and 4, ≥31 root knots, mostly continuous without fine roots. *M. incognita* J2s were isolated from 20 g of soil collected from each pot after 48 h using the Baermann funnel method (26) and then counted, with a total of 3 replicates per soil sample.

## Results

### Effects of BPE on the hyphal growth of nematode-trapping fungi

The hyphal growth of *A. dactyloides*, *A. oligospora*, and *M. ellipsosporum* with all extract treatments was significantly less than that with the control, and the inhibitory effects of the 10-fold dilution of the extract was particularly strong. In contrast, *M. eudermatum* hyphal growth relative to the control was inhibited by all treatments, except the 100-fold dilution, whereas the growth of *M. phymatopagum* hyphae was significantly inhibited from that by the control only by the 10-fold dilution (Table 1).

### Effects of BPE on the formation of capture organs and the nematode-trapping rate

The highest number of capture organs formed was observed with *A. dactyloides* treated with the stock solution and 10-fold dilution, at 438.8 and 117.2, respectively. By comparison, *A. oligospora* formed 87.0 and 36.0 capture organs following the treatment with the stock solution and 10-fold dilution, respectively. The number of capture organs formed by *A. dactyloides* and *A. oligospora* was higher than that formed by the other species examined. Very few capture organs (0.0–0.2) were formed by *M. ellipsosporum*, *M. eudermatum*, and *M. phymatopagum* following the same treatments (Table 2). With respect to the effects of the extracts on the nematode-trapping rate, *A. dactyloides* showed a 100% trapping rate after the treatment with the stock solution, whereas *A. oligospora* showed a 90.9% trapping rate. The trapping rates of *A. dactyloides* and *A. oligospora* were also high for the treatment with the 10-fold dilution at 32.5 and 29.0%, respectively. In contrast, *M. ellipsosporum*, *M. eudermatum*, and *M. phymatopagum* exhibited no nematode trapping (Table 3).

### Effects of the formulation composed of BPE and *A. dactyloides* on the formation of capture organs and the nematode-trapping rate

None of the treatments significantly affected the formation of capture organs 1, 3, or 5 d after the nematode inoculation 0.3–0.7%, 33.8–34.2%, and 36.3–39.3%, respectively, and treatments were approximately equal 3 d after the nematode inoculation.

No significant effects were observed on the nematode-trapping rate (0.3–0.5%) 1 d after the nematode inoculation, whereas the trapping rate of the simple formulation (stock solution: 89.8, 10-fold dilution: 80.6) 5 d after the inoculation was higher (Table 4).

### Efficacy of the simple mixed formulation for controlling *M. incognita*

No significant differences were observed in the plant height, shoot weight, or root weight of tomato plants between any treatment and the control. However, a significant increase in root length was observed for plants treated with the mixed formulation composed of fungus and BPE (stock solution) (Table 5).

Regarding the root-knot index, the highest value (3.7) was observed for the control. Moreover, the root-knot index of the mixed formulation and treatments composed only of the fungus or BPE exhibited efficacy (0.6–2.7), with the mixed formulation composed of the fungus and stock solution of BPE showing the highest efficacy for controlling *M. incognita* (Table 5).

## Discussion

A number of organisms are known to prey on or infect plant-parasitic nematodes, including arthropods, predacious nematodes, mites and springtails, bacteria, actinomyces, and fungi (5, 27). In addition, several microbial-based anti-nematode products have been developed, including products containing *M. phymatopagum* or *P. penetrans* as the main ingredient (42). Moreover, research has also focused on the use of plant extracts against nematodes, and the use of weeds, medicinal herbs, and seaweeds against nematodes has been reported (2, 5, 16, 17, 22, 43) because they exhibit nematicidal activities or inhibit the hatching of harmful nematodes. In addition to the use of plant extracts alone, the combined use of plant extracts and predatory or endo-parasitic fungi (21, 24) or the pomace of seeds and whole plants (*e.g.*, neem cake and antagonistic plants) has been described (1, 5). Quarles *et al.* (23) showed that plant extracts have a number of important characteristics: they provide novel compounds that pests cannot inactivate; these active compounds are less concentrated and, thus, potentially less toxic than pure compounds; the active compounds biodegrade rapidly and may possess multiple modes of action, making a wide spectrum of use possible while retaining selectivity within each pest class; and since they are derived from renewable resources, these compounds may represent a new class of materials for environmentally beneficial pest control.

A previous study demonstrated that *B. pilosa* var. *radiata* exhibits nematicidal activity against plant-parasitic nematodes (38); therefore, the present study was conducted in order to investigate the effectiveness of combining BPE with nematode-trapping fungi. The limited inhibition of hyphal growth by nematode-trapping fungi was observed with the plant extracts (Table 1). However, experiments examining the formation of capture organs, particularly those of *A. dactyloides*, which formed rings whose numbers were approximately 11-fold more than that by the control (Tables 2 and 3), revealed that the nematode-trapping rate increased with the addition of BPE. Gronvold *et al.* (8) and Sun and Liu (31) reported that the formation of capture organs is dependent on the species and type of fungus as well as biological and abiological environmental characteristics. Moreover, Balan and Lechevalier (3) found that the formation of capture organs in *A. dactyloides* was induced by athrepsy or dehydration. Based on the results obtained in this study, we hypothesized that the inhibition of fungal growth by the antimicrobial substances contained in BPE made it difficult for fungi to acquire sufficient nutrients from the medium, which resulted in an increase in the formation of capture organs for nourishment acquisition. Although plant extracts reportedly inhibit the growth of bacteria and fungi (4, 9), in the case of *A. dactyloides*, this may be an advantageous condition for a biological control agent because the nematode-trapping ability of the fungus improved while hyphal growth was inhibited.

The results of survival tests on *A. dactyloides* and the effects of the simple formulation on nematode trapping were the absence of a decrease in fungal survival (Table 4). In order to produce a microbiological agent incorporating a fungus as an ingredient, it is important to investigate the availability of a mass culture and the effects of fixability, humidity, ultraviolet rays, dryness, preservability, and agricultural chemicals on the activity of the agent (11, 12). Moreover, nematode-trapping fungi are known to be sensitive to bacteriostasis (15, 40), and, thus, fixability in soil and the establishment of an efficient use period and method are considered to be important.

In the pot test, a suppressive effect on root-knot formation was observed with the fungus only, BPE only, and both mixed formulations, with the mixed formulations exerting the greatest effects. Therefore, the mixing of BPE and fungus is considered to be the primary factor influencing the efficacy of treatment. Previous studies described simultaneous treatments with plant extracts and nematode-trapping or parasitic (endo-parasitic) fungi (21, 24); however, there are no examples demonstrating highly effective control through independent treatments with a mixture. Taba *et al.* (37) showed that a simultaneous treatment with an agricultural chemical and nematode-trapping fungus (*M. ellipsosporum*) was an effective means of reducing the use of pesticide chemicals. However, combining two materials with biological origins is an eco-friendly control method because the potential impact on the environment is reduced further. Although considerable research has focused on controlling harmful nematodes using nematode-trapping fungi, the findings obtained to date have been inconsistent (7, 10, 13, 15, 19, 28, 29, 30). Therefore, future studies on formulations are needed that focus on stabilizing and improving control by clarifying the interactions not only between nematodes and fungi, but also between nematodes, soil, and plants.

In the present study, the nematicidal effects of mixed formulations of BPE and nematode-trapping fungi were characterized. Since the effects of a biological control method are generally influenced by a number of environmental factors, sufficient effects cannot be demonstrated in many cases. Therefore, integrated pest management approaches combined with other environmentally conscious control methods are important.

## Figures and Tables

**Table 1:** Effects of *Bidens pilosa* var. *radiata* aqueous extract on the hyphal growth of nematode-trapping fungi

Species (capture organ)	Colony diameter (cm)					

	Stock solution1)	102)	5	2	1	Control3)
*Arthrobotrys dactyloides* (ring)	6.4±0.1 bc4)	4.9±0.1 f	5.6±0.2 e	6.1±0.1 d	6.5±0.2 b	7.5±0.2 a
*A. oligospora* (network)	7.1±0.1 b	5.7±0.2 e	6.2±0.4 c	6.9±0.1 b	7.0±0.2 b	7.7±0.1 a
*Monacrosporium ellipsosporum* (knob)	6.5±0.3 b	5.6±0.2 c	5.9±0.1 bc	6.3±0.2 bc	6.5±0.3 bc	7.3±0.2 a
*M. eudermatum* (network)	7.1±0.2 b	6.7±0.0 bc	6.9±0.1 b	7.1±0.1 b	7.4±0.2 a	7.6±0.1 a
*M. phymatopagum* (ladder to network)	7.1±0.2 ab	7.0±0.2 b	7.3±0.2 ab	7.4±0.1 a	7.4±0.1 a	7.6±0.2 a

1) Stock solution prepared from 10 g dry plant material 50 mL^−^^1^ water.

2) Diluted concentration (%)

3) Sterilized distilled water.

4) Colony diameters (mean±SD, six replicates) measured, *A. dactyloides* 19 d, *A. oligospora* 7 d, *M. ellipsosporum* 12 d, *M. eudermatum* 5 d, *M. phymatopagum* 7 d after the inoculation are presented.

Values in a row with the same letters are not significantly different according to Tukey’s HSD multiple comparison test, *P*<0.05.

**Table 2:** Effects of *Bidens pirosa* var. *radiata* aqueous extract on the formation of capture organs of nematode-trapping fungi

Species (capture organ)	Stock solution1)	10-fold dilution	Control2)
*Arthrobotrys dactyloides* (ring)	438.8±79.2^**^3)	117.2±33.9	39.3±18.1
*A. oligospora* (network)	87.0±16.5^**^	36.0±10.7^*^	11.0±3.5
*Monacrosporium ellipsosporum* (knob)	0.0±0.0	0.0±0.0	0.2±0.4
*M. eudermatum* (network)	0.0±0.0	0.2±0.4	0.5±0.8
*M. phymatopagum* (ladder to network)	0.0±0.0	0.0±0.0	0.0±0.0

1) Stock solution prepared from 10 g dry plant material 50 mL^−1^ water.

2) Sterilized distilled water.

3) Mean±SD of 6 replicates.

Data in the table are shown 7 d after the inoculation. The asterisks indicate a significant difference from the control group (Dunnett’s multiple comparison test, symbols: ^**^ *P*<0.001; ^*^ *P*<0.01).

**Table 3:** Effects of *Bidens pilosa* var. *radiata* aqueous extract on the nematode-trapping rate (%)

Species (capture organ)	Stock solution1)	10-fold dilution	Control2)
*Arthrobotrys dactyloides* (ring)	100.0±0.0**3)	32.5±11.4	16.9±16.0
*A. oligospora* (network)	90.9±3.3**	29.0±11.2	15.6±12.4
*Monacrosporium ellipsosporum* (knob)	0.0±0.0	0.0±0.0	2.4±5.8
*M. eudermatum* (network)	0.0±0.0	0.0±0.0	1.9±2.9
*M. phymatopagum* (ladder to network)	0.0±0.0	0.0±0.0	0.0±0.0

1) Stock solution prepared from 10 g dry plant material 50 mL^−1^ water.

2) Sterilized distilled water.

3) Mean±SD of 6 replicates.

Data in the table are shown 7 d after the inoculation. The asterisks indicate a significant difference from the control group (Dunnett’s multiple comparison test, symbols: * *P*<0.001).

**Table 4:** Effects of the formulation containing a mixture of *Bidens pilosa* var. *radiata* extract and *Arthrobotrys dactyloides* on the formation of capture organs and the nematode-trapping rate

Treatment	Number of capture organs (4 cm in diam.)			Nematode-trapping rate (%)		
	
	1	3	5 (d)	1	3	5 (d)
Fungus+extract (Stock solution)1)	0.7±1.0 a2)	34.2±9.5 a	36.3±5.3 a	0.5±0.9 a	43.2±6.8 a	89.8±7.6 a
Fungus+extract (10-fold dilution)	0.3±0.5 a	33.8±5.5 a	39.3±6.7 a	0.3±0.5 a	55.7±9.5 a	80.6±6.6 a

1) Stock solution prepared from 10 g dry plant material 50 mL^−1^ water.

2) Mean±SD of 6 replicates.

Different letters in the same vertical column indicate a significant difference (Tukey’s HSD multiple comparison test, *P*<0.05).

**Table 5:** Control efficacy of the mixed formulation containing *Bidens pilosa* var. *radiata* aqueous extract (BPE) and *Arthrobotrys dactyloides* on the growth of tomato and root-knot formation by *Meloidogyne incognita*

Treatment	Plant height (cm)	Shoot weight (g)	Root length (cm)	Root weight (g)	Root knot index
BPE1)+Fungus2)	18.7±3.8	7.4±1.8	26.7±8.6*4)	3.0±0.6	0.6±0.4 d5)
BPE only	16.6±1.6	5.5±0.6	19.8±1.0	2.0±0.2	1.9±0.2 c
Fungus only	17.1±5.1	5.3±3.3	16.4±5.1	1.8±1.0	2.7±0.3 b
Control3)	17.8±4.3	6.2±3.1	14.2±4.7	1.9±0.8	3.7±0.3 a

1) Stock solution prepared from 10 g dry plant material 50 mL^−1^ water.

2) Separated partial hyphae by the centrifugation (2.4×10^5^ mL^−1^) of *A. dactyloides*.

3) Without formulations (mixed and non-mixed), but with the *M. incognita* inoculation.

4) The asterisks indicate a significant difference from the control group (Dunnett’s multiple comparison test, *P*<0.01).

5) Different letters in the same vertical column indicate a significant difference (Steel-Dwass test, *P*<0.05).
